# Targeted Positioning of Quantum Dots Inside 3D Silicon Photonic Crystals Revealed by Synchrotron X-ray Fluorescence Tomography

**DOI:** 10.1021/acsnano.1c06915

**Published:** 2022-02-21

**Authors:** Andreas S. Schulz, Cornelis A. M. Harteveld, G. Julius Vancso, Jurriaan Huskens, Peter Cloetens, Willem L. Vos

**Affiliations:** † Complex Photonic Systems (COPS), MESA+ Institute for Nanotechnology, University of Twente, P.O. Box 217, 7500 AE Enschede, The Netherlands; ‡ Molecular Nanofabrication (MNF), MESA+ Institute for Nanotechnology, University of Twente, P.O. Box 217, 7500 AE Enschede, The Netherlands; § Materials Science and Technology of Polymers (MTP), MESA+ Institute for Nanotechnology, University of Twente, P.O. Box 217, 7500 AE Enschede, The Netherlands; ∥ ESRF-The European Synchrotron, CS40220, 38043 Grenoble, France

**Keywords:** 3D integration, complementary metal-oxide-semiconductor (CMOS), nanofabrication, photonic crystals, quantum dots, X-ray fluorescence imaging

## Abstract

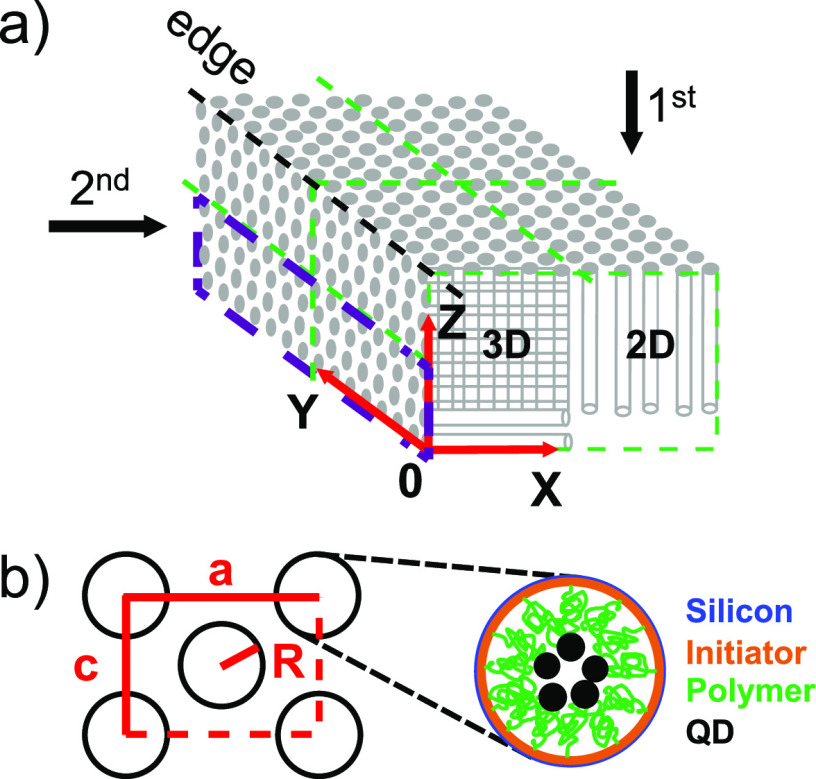

It is a major outstanding goal in nanotechnology to precisely position functional nanoparticles, such as quantum dots, inside a three-dimensional (3D) nanostructure in order to realize innovative functions. Once the 3D positioning is performed, the challenge arises how to nondestructively verify where the nanoparticles reside in the 3D nanostructure. Here, we study 3D photonic band gap crystals made of Si that are infiltrated with PbS nanocrystal quantum dots. The nanocrystals are covalently bonded to polymer brush layers that are grafted to the Si–air interfaces inside the 3D nanostructure using surface-initiated atom transfer radical polymerization (SI-ATRP). The functionalized 3D nanostructures are probed by synchrotron X-ray fluorescence (SXRF) tomography that is performed at 17 keV photon energy to obtain large penetration depths and efficient excitation of the elements of interest. Spatial projection maps were obtained followed by tomographic reconstruction to obtain the 3D atom density distribution with 50 nm voxel size for all chemical elements probed: Cl, Cr, Cu, Ga, Br, and Pb. The quantum dots are found to be positioned inside the 3D nanostructure, and their positions correlate with the positions of elements characteristic of the polymer brush layer and the ATRP initiator. We conclude that X-ray fluorescence tomography is very well suited to nondestructively characterize 3D nanomaterials with photonic and other functionalities.

## Introduction

Three-dimensional (3D) functionalized nanostructures are drawing fast-growing attention for their advanced applications in nanophotonics,^1−3^ photovoltaics,^4,5^ capacitors in electronics,^6^ gas sensing,^7^ materials for electrochemical energy conversion and storage,^8^ and batteries.^9−12^ The functionalization of these nanostructures is the result of the infiltration of active nanoparticles, e.g., fluorophores in nanophotonic light sources,^13−15^ antibodies for biochemical sensors of diseases, or quantum dots for photovoltaics.^16,17^ In most cases, the performance of the 3D functionalized nanostructure depends on the precise positioning of the nanoparticles inside the 3D nanostructure, with nanometer precision. In nanophotonics, for instance, fluorescing nanoparticles should reside at positions where the local density of optical states is either maximal, in the case of cavities or antennae,^18−20^ or minimal in the case of a photonic band gap.^21,22^ In biochemical detectors, antibodies should be positioned on the internal interfaces of the 3D substrate for maximum reactivity and selectivity.^23^

## Results and Discussion

**Figure 1:**
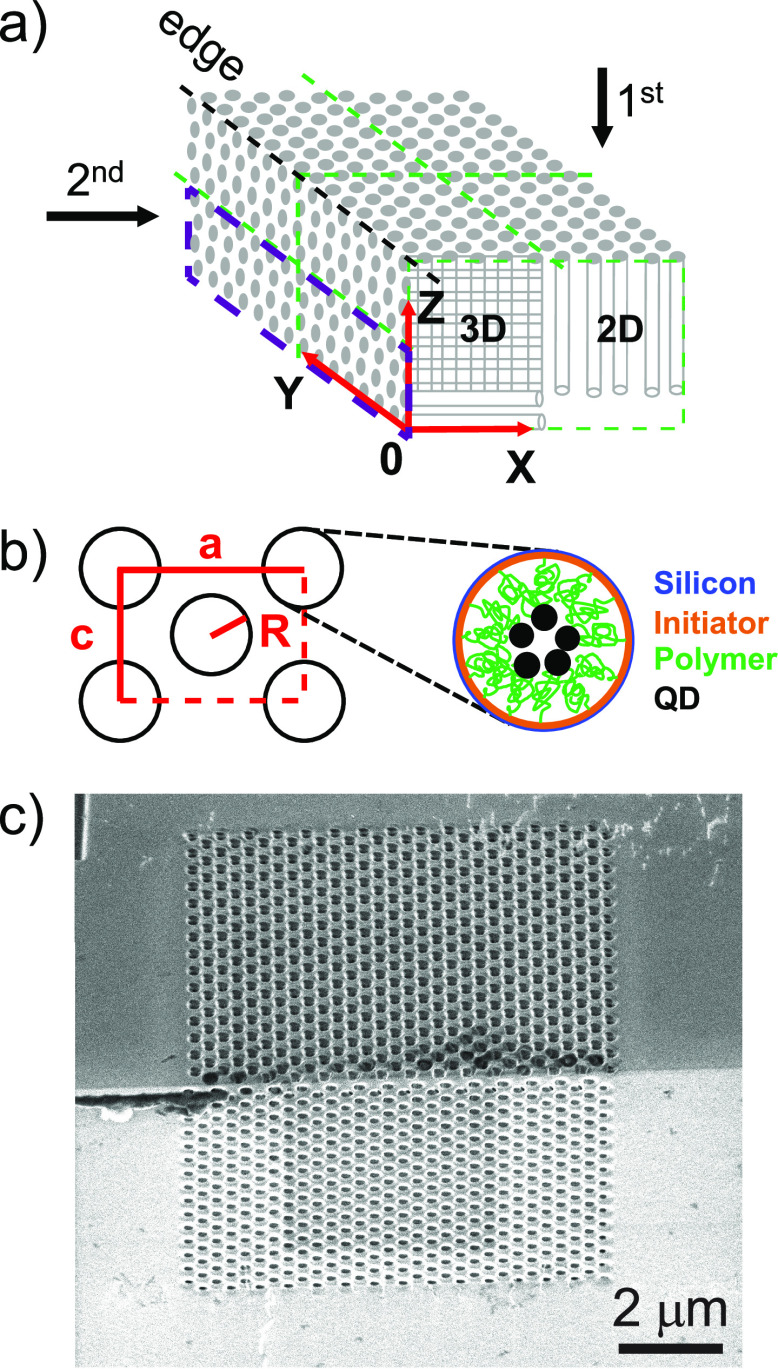
3D photonic crystal and chemical positioning of quantum dots inside of the nanostructure. (a) Schematic of an inverse woodpile photonic crystal on the edge of a silicon beam. Green dashed lines indicate the two surfaces into which nanopores are etched. The 3D and 2D parts of the crystal are indicated in the *XZ* front face. (b) View along the pores showing the lattice parameters *a*, *c* (with ), and pore radius *R*. Zoomed-in cross-section of one pore with targeted surface-chemistry: ATRP initiator layer (orange), polymer chains forming brushes (green), and covalently attached PbS quantum dots (black) on top of silicon (blue). (c) Scanning electron microscope (SEM) image of a 3D photonic crystal viewed from 45° on the edge of the silicon beam showing the *XY* (top) and the *XZ* surfaces (bottom); the scale bar indicates 2 μm.

In this representative study, we fabricate 3D photonic band gap crystals from silicon with the diamond-like inverse woodpile structure consisting of two perpendicular arrays of interpenetrating nanopores,^24^ see Figure 1a. We infiltrate functional quantum dot nanoparticles in these crystals that are precisely positioned relative to the silicon–air interfaces by means of polymer brushes^25,26^ that grow from initiator molecules placed on the internal silicon interfaces, see Figure 1b. In previous photonic band gap crystal studies, quantum dots were randomly positioned in the crystal,^3^ whereas brushes will allow selective positioning of the dots at places where they experience the maximal band gap effects.^22^ Once the nanoparticles have been infiltrated, the challenge addressed here is to find their final positions. Therefore, the first requirement is to find a probing method with the first requirement that it provides local information deep inside a 3D nanostructure with nanometer spatial resolution. A second requirement is that the method is element specific to verify that the infiltrated nanocrystals are located in the desired positions and to characterize the infiltration mechanism or process. A third major requirement is that the probing technique must be nondestructive, hence the nanomaterial remains functional and ready for further integration after the inspection. While the widely used scanning electron microscopy (SEM) nondestructively reveals the periodically ordered external surfaces of the photonic crystal (Figure 1c), internal nanoparticles are hidden, even at a much higher resolution.

A common approach in nanotechnology to view elements inside 3D nanostructures is to cleave or ion-mill the sample and then probe the exposed surface with energy dispersive X-ray spectroscopy,^27^ time-of-flight secondary-ion mass spectrometry,^28^ X-ray photoelectron spectroscopy,^29^ or Fourier-transform infrared or Raman spectroscopy.^30^ A major disadvantage of this approach is that it is destructive. Moreover, several of the probing methods have coarse spatial resolutions in the range of 10 μm or even coarser. X-ray techniques offer suitable nondestructive analysis methods due to their high penetration and high resolution.^31,32^ Anomalous small-angle scattering of X-rays allows the obtaining of contrast variation in a multicomponent system.^33,34^ Unfortunately, the number of elements that can be distinguished is limited because many incident X-ray wavelengths have to be independently tuned, and it is very difficult to obtain local information from such a scattering method. Here, we demonstrate the use of X-ray fluorescence tomography using a synchrotron source^35−37^ that meets all above requirements, to inspect designed functional nanostructured samples.

**Figure 2:**
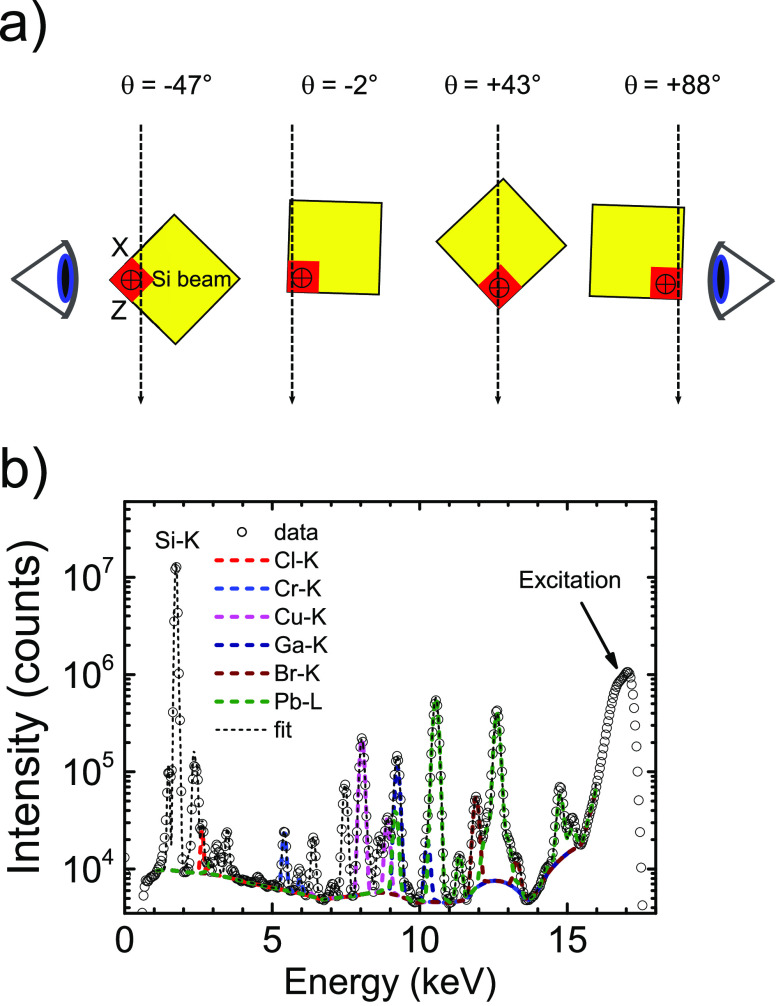
Sample mounting and X-ray fluorescence spectra. (a) Top view showing how the X-ray beam traverses the sample on the Si substrate at four different angles θ. Two detectors collect X-ray fluorescence signal on the left and right sides. (b) X-ray fluorescence spectrum *I*(*E*, θ) (circles) of one of the crystals averaged over all 240 × 240 pixels in the image at θ = −47°. The analyzed Cl, Cr, Cu, Ga, Br, and Pb transitions are shown in order of increasing photon energy.

In X-ray fluorescence tomography, the sample is illuminated with a finely focused X-ray beam to excite X-ray fluorescence that is characteristic for each element in the sample, see Figure 2a. At θ = −47°, the incident beam passes obliquely through the crystal. The right detector will detect the fluorescence signal that is integrated along the incident X-ray beam (the left detector will detect little signal as it is attenuated by the substrate.) At θ = −2°, the incident beam traverses the whole 500-μm-thick Si substrate, which is feasible on account of the high photon energy of 17 keV. The detected fluorescence signal is integrated along the *X* direction, hence the left detector effectively sees a projection of the object in the *YZ* plane. At θ = 43°, the incident beam first passes through even more substrate thickness (by projection) before entering the photonic crystal. Both detectors see an oblique projection of the sample. At θ = 88°, the X-ray beam travels nearly in the high-symmetry −*Z* direction; the (left) detector detects fluorescence integrated along the −*Z* direction and thus effectively detects a projection of the object in the *XY* plane.

Figure 2b shows a typical X-ray fluorescence spectrum that is averaged over all 240 × 240 pixels. We identify the involved elements from their known X-ray emission with constraints from the known chemical compounds involved in the sample preparation (see Methods). The peak at 1.74 keV is the Si Kα transition from the photonic crystal matrix. In order of increasing photon energy, chlorine, chromium, copper, gallium, bromine, and lead are detected. Chlorine features in the Cu(I)Cl species used as a catalyst in the polymerization and in the termination group of the polymer brushes (see Figure 1b). Chromium appears in the sample as the hard-mask material for the deep reactive-ion etching of the pores that define the 3D photonic crystal. Copper is introduced by the two catalyst species Cu(I)Cl and Cu(II)Br that are incompletely washed out due to the limited permeability of such a complex 3D porous nanostructure. Gallium occurs since it is used to write the etch mask by means of focused-ion beam milling. Bromine features in the ATRP-initiator monolayer for the brush synthesis and also in the Cu(II) catalyst species used in the polymerization (see Figure 1b). Finally, lead is the signature of the infiltrated PbS quantum dots with a strong signal since it is a heavy element. We note that sulfur from the quantum dots is not detected, since the signal is attenuated due to the low photon energy and is likely overwhelmed by the strong Si Kα peak at 1.74 keV. The peak at 17 keV is the excitation by the incident X-ray beam that is broadened due to Compton scattering and a finite detector resolution.

**Figure 3:**
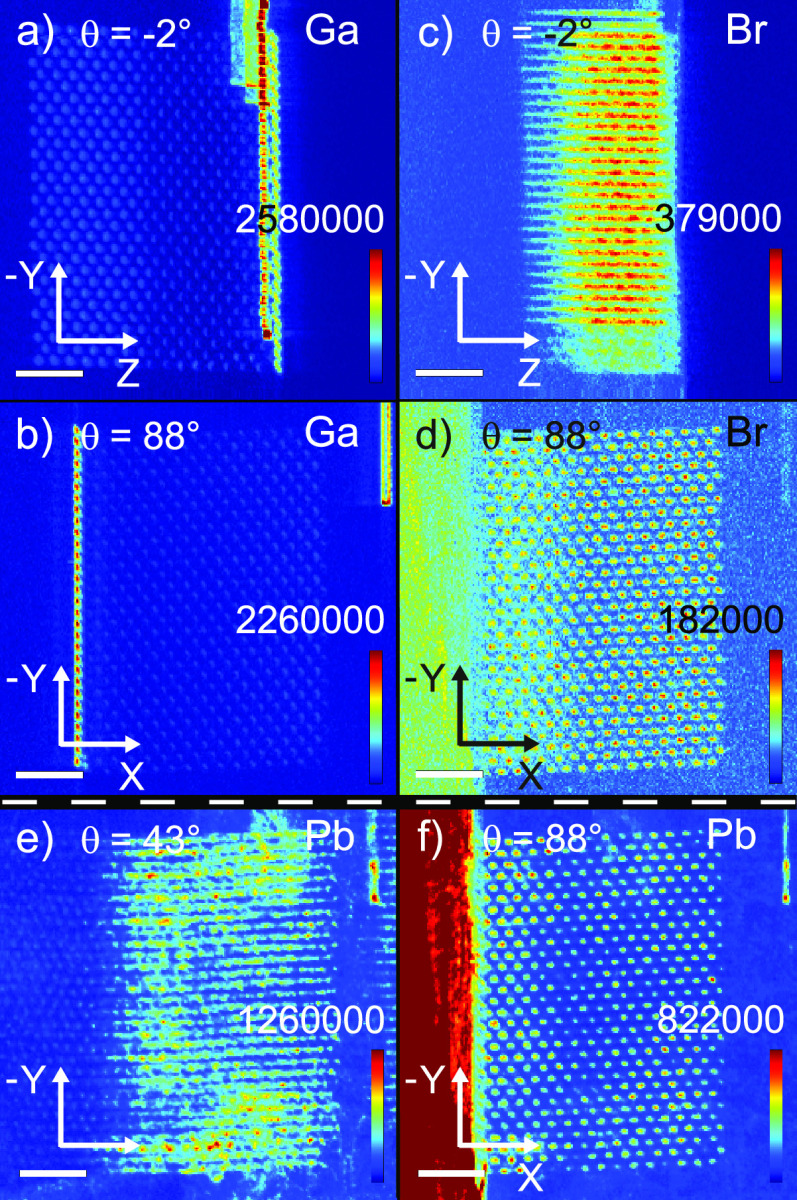
Projection maps of the number of Ga, Br, and Pb atoms per pixel (with area 50 × 50 nm^2^), integrated along the propagation direction of the beam, for sample orientations: (a) Ga at θ = −2°, (b) Ga at θ = 88°, (c) Br at θ = −2°, (d) Br at θ = 88°, (e) Pb at θ = 43°, (f) Pb at θ = 88°. Color bars with the density scales are given in each panel.

At every sample orientation θ (Figure 2a), the X-ray focus is raster scanned through the crystal (see Methods). The X-ray fluorescence signals separated for all elements yield so-called projection maps. These are 2D maps of the elemental number density per pixel area integrated along the X-ray beam, while it traverses the sample. Figure 3 shows examples for Ga and Br at two different orientations θ.

Figure 3a shows the gallium signal projected on the *YZ* plane. From left to right, we see (faintly) the nanopore entrances (on the *YZ* crystal surface) that are defined in the lithography step by focused-ion beam milling using Ga ions. At two-thirds from the left, there is a bright streak of the Ga signal in the *Y* direction, because the fluorescence signal is added (projected) in the *X* direction for all nanopore entrances in the *XY* crystal surface. Figure 3b shows the gallium signal projected on the *XY* plane. At one-fourth from the left, there is a bright streak of Ga signal in the *Y* direction, because the fluorescence signal is added (projected) in the *Z* direction for all nanopore entrances in the *YZ* crystal surface. Further to the right, we (faintly) see the nanopore entrances on the *XY* crystal surface. These observations confirm that the gallium atoms are effective markers of the external crystal surface.

In Figure 3c, we observe the Br density in the photonic crystal from a high-symmetry angle viewing along the *X* axis onto the *YZ* plane. The Br density is arranged in horizontal stripes, due to the *Z* directed pores. Therefore, we conclude that Br is present throughout the whole extent of the nanopores. Thus, the ATRP-initiator has infiltrated the pores of the 3D silicon photonic crystal to form a monolayer throughout the whole structure (based on previous results on flat Si wafers^26^), which is crucial for further functionalization of the surface inside the 3D silicon crystal pores. It is also conceivable that part of the Br density is caused by incompletely rinsed Cu(II)Br catalyst that is used in the synthesis. In both cases, we conclude that intended chemistry has taken place inside the nanopores.

In Figure 3d, we observe the projection in the *XY* plane that shows the periodicity of the nanopores as individual disks of Br atoms. We do not observe thin circular shapes as expected in the case of purely surface deposition on perfectly cylindrical pores. Since the pores are not perfectly perpendicular to the XY plane and have a depth-dependent diameter (known as tapering^38^), the projected Br density appears as filled ellipses. On the left side, the edge of the silicon beam is visible.

Figure 3e shows the lead signal projected at θ = 43°, which shows a diagonal projection of the crystal (compare Figure 2a). The projection shows that the Pb atoms (and thus the quantum dots) form horizontal stripes, just the nanopores at such a projection. Therefore, this is a first indication that the Pb atoms have infiltrated the 3D nanostructure.

In Figure 3f, we observe the projection in the *XY* plane that shows the periodicity of the nanopores as disks of Pb atoms. The high density on the left side of the image is caused by the incident beam being nearly parallel to the sample surface (compare Figure 2a) and thereby exciting many Pb atoms on the whole external surface of the Si sample.

Projection maps are collected while rotating the sample by 180°, see Figure 2a. After tomographic reconstruction (see Methods), one obtains a position-resolved 3D density map of all elements that are detected inside the nanostructure. In the case of Cl and Cr, the signals were relatively weak (only a few times 10^4^ counts after background subtraction) so that their projection maps were relatively noisy, hence no firm conclusions could be drawn on the positions of these elements.

**Figure 4:**
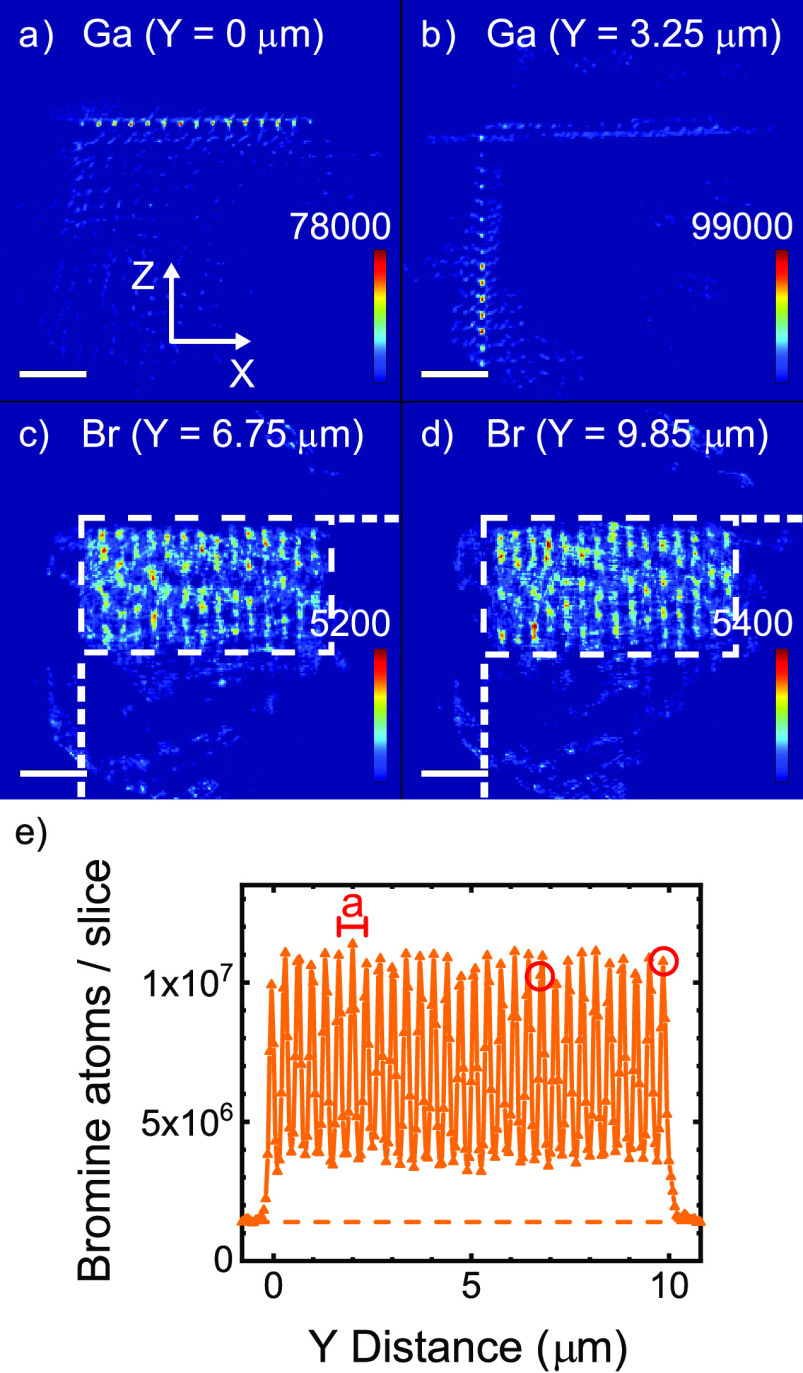
*XZ* cross sections of the Ga atomic density at depths *Y* = 0 μm (a) and *Y* = 3.25 μm (b). *XZ* cross sections of the Br atomic density at *Y* = 6.75 μm (c) and *Y* = 9.85 μm (d). The scale bars represent 2 μm; the intensity bars, the number of atoms per voxel (with volume 50^3^ nm^3^). White dashed lines delineate the crystal’s cross section and the external surface. (e) Number of bromine atoms per slice integrated along *Z*, as a function of *Y*. The lattice parameter *a* of the photonic crystal is shown. The periodicity of the pores is clearly visible. Red circles indicate the *Y* coordinates in c and d.

Figure 4a,b show *XZ* cross sections of the 3D density map of gallium *N*_v,Ga_(*X*,*Y*,*Z*) (the whole data set is shown as a *Y* scan of *X*–*Z* cross sections in Supporting Information S2). The gallium is located near the external surfaces of the photonic crystal, which is reasonable since Ga ions are used to define the etch masks for the nanopores on the external surfaces of the Si beam substrate by focused-ion beam milling.^39^ Since there is hardly any Ga signal from the bulk of the Si substrate, there are few artifacts due to the tomographic image reconstruction, which confirms that the reconstruction has converged well. Figures 4c,d show *XZ* cross-sections of the 3D density map of bromine atoms *N*_v,Br_ (*X*,*Y*,*Z*), see Figure 1b. The Br density is distributed in vertical stripes in the *Z* direction, showing that the Br atoms have infiltrated the nanopores, to as deep as Δ*Z* = 3.5 μm. All *Z*-directed nanopores are filled from the sample surface, since all pores over an *X*-extent Δ*X* = 7.2 μm reveal similar Br density profiles. We also observe that Br atoms are infiltrated in the *X*-directed pores at intermediate *Y* coordinates (see supplementary movie S1). Thus, the Br atoms effectively outline the nanopore structure in the silicon. These observations match our expectations because the initiator molecules were introduced as a vapor that readily diffuses into a porous sample. Therefore, the silane monolayer due to the ATRP deposition method resides on the surfaces of the nanopores. The presence and homogeneity of the silane monolayer influences and defines the subsequent functionalization with the polymer brush layer.

Figure 4e shows the total number of Br atoms per *XZ* slice as a function of *Y* position. We observe that the bromine density shows a sine-like profile in the whole crystal between *Y* = 0 and 10 μm. The period is equal to *a*/2, which is sensible because the fluorescent signal from inside the pores is observed *twice* per unit cell since there are two planes of pores within each unit cell, as shown in Figure 1b. We observe that the maximum amplitude is constant to within 13% and the minimum amplitude is constant to within 21%, confirming a fairly homogeneous infiltration. The open red circles mark the *Y* positions of the slices in Figure 4c,d. We therefore conclude that the upper amplitude represents the bromine densities in the pores in the *Z* direction, whereas the lower amplitude represents the bromine densities in the pores in the *X* direction. The amplitude oscillates between 3.6 × 10^6^ and 0.8 × 10^7^ bromine atoms per slice, whereas the finite density outside the crystal is 1.3 × 10^6^ bromine atoms per slice. When we correct the densities in the crystal for this background, the density in the pores in the *Z* direction equals (9.4 × 10^6^)/(2.2 × 10^6^) = 4.3× greater than in the pores in the *X* direction. One possible explanation for the different average Br densities is that the pores in the *X* direction were etched in the second etching step with the presence of fluorocarbons on the *YZ* surface. It is then conceivable that these fluorocarbons that were used in the protection steps of the etching process were incompletely removed by the oxygen plasma cleaning in the etching machine after the etching of the first set of pores in the *Z* direction, thereby (partly) blocking the infiltration of these nanopores.

**Figure 5:**
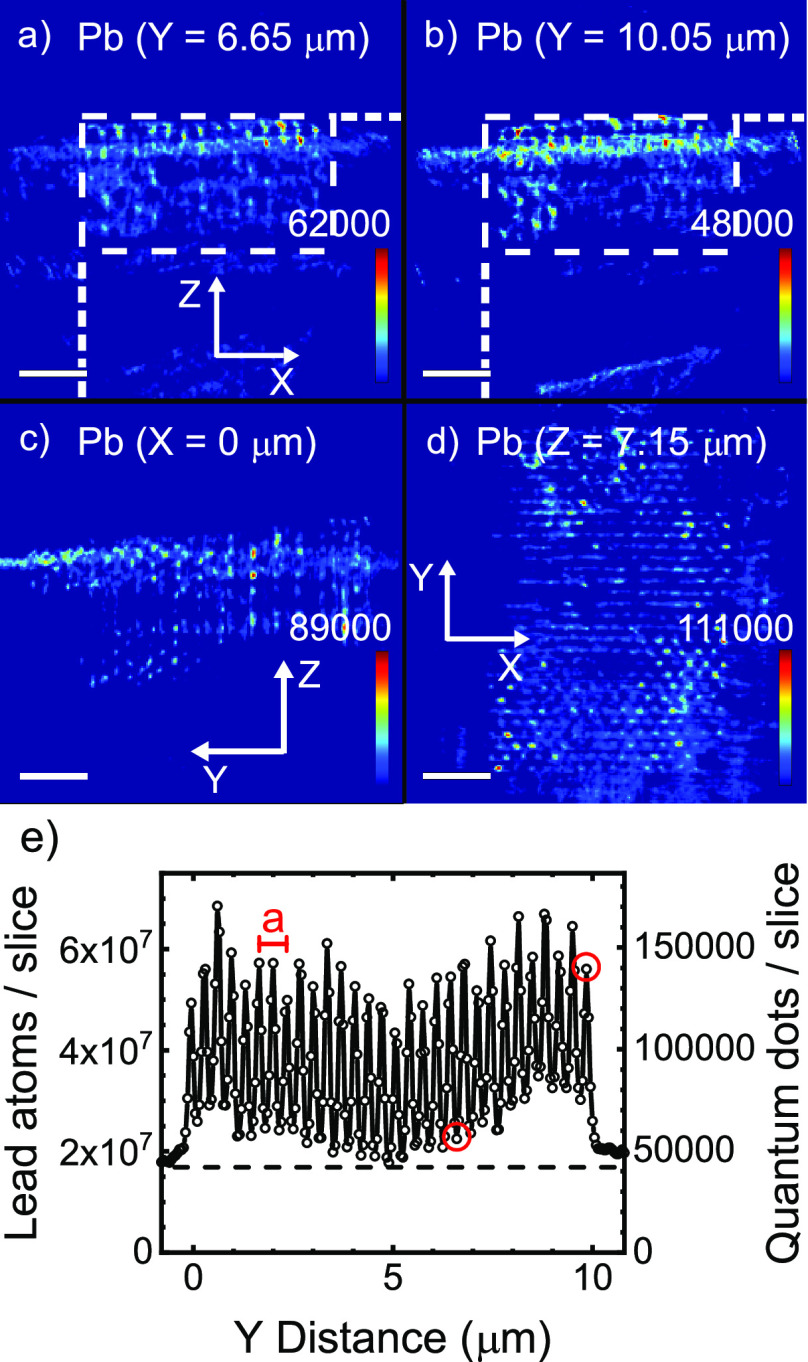
(a,b) *XZ* cross sections of the reconstructed sample volume for the Pb atoms at depths *Y* = 6.65 μm (a) and *Y* = 10.05 μm (b). The scale bar represents 2 μm; the intensity bar, the number of lead atoms per voxel; and white dashed lines delineate the crystal’s cross-section. (c) *YZ* cross-section of the reconstructed sample volume for the Pb atoms at depth *X* = 0 μm. (d) *XY* cross-section of the reconstructed sample volume for the Pb atoms at depth *Z* = 7.15 μm. (e) Density of lead atoms per *XZ* slice versus *Y*; the right ordinate shows the corresponding number of quantum dots (taking 402 Pb atoms per dot). The lattice parameter *a* of our photonic crystal is indicated. The periodicity of the pores is clearly visible. The red circles show the *Y* coordinates in a and b.

Figure 5a,b show *XZ* cross-sections of the 3D density map of lead atoms *N*_v,Pb_(*X*,*Y*,*Z*), which are the cations of the infiltrated PbS quantum dot nanoparticles, see Figure 1b. The Pb density extends along the pores in the *Z* direction, starting from the *XY* top surface and extending about 3.5 μm into the crystal. Figure 5c show a *YZ* cross-section of the 3D density map of lead atoms *N*_v,Pb_(*X*,*Y*,*Z*). The quantum dots extend along the pores in the *Z* direction, starting from the *XY* top surface and extending about 3.5 μm into the crystal (the whole data set is shown as a *Y* scan of *X*–*Z* cross sections in Supporting Information S4).

Figure 5d shows a *YX* cross-section of the 3D density map of lead atoms *N*_v,Pb_(*X*,*Y*,*Z*). The pores are visible as small dots on the *XY* surface. We conclude that the quantum dots have indeed been successfully infiltrated into the nanopores of the 3D photonic crystal.

To characterize in more detail the infiltration of the quantum dots in each nanopore, we plot in Figure 5e the density of lead atoms integrated along *Z* as a function of the *Y* coordinate. The Pb density oscillates throughout the whole crystal between *Y* = 0 to 10 μm with a period equal to *a*/2, as with the Br data in Figure 4e, due to two planes of nanopores per unit cell. The Pb density amplitude in Figure 5e oscillates between 1.8 × 10^7^ and 6.8 × 10^7^ lead atoms per slice. Outside the crystal, the lead density is between 1.8 × 10^7^ and 2.1 × 10^7^ atoms/(50 nm × 50 nm × 3.5 μm), which is mostly determined by quantum dots on the external sample surface (a real signal) and by artifacts due to the 2D structure that lies behind the 3D structure. At *Y* = 5 μm, the Pb density modulation is somewhat reduced before increasing again toward the right edge of the crystal at *Y* = 10 μm.

Since we know from the Br density (see Figure 4c,d) that the *Z*-directed nanopores have a depth of 3.5 μm, the exhilarating conclusion arises that the quantum dots have been successfully infiltrated over the full depth of the nanopores. Similar to Br at other *Y* coordinates (see supplementary movie S2), we also observe Pb over the full depth of the *X*-directed pores. Therefore, our strategy to infiltrate quantum dots inside 3D nanostructures by attaching them to polymer brushes has been successful. Moreover, this result demonstrates that X-ray fluorescence tomography is a powerful method to characterize the infiltration of functional nanoparticles in complex 3D architectures.

We observe that the maximum amplitude in Figure 5e is constant to within 37%, and the minimum amplitude is constant within 50%. These amplitudes for the lead atoms are less homogeneous than for bromine, which is reasonable for three reasons. (i) The Br atoms originating from the initiator molecules are introduced in the vapor phase, hence the diffusion is the fastest, leading to the most homogeneous infiltration. Moreover, the rinsing of excess molecules of this species also likely has the most homogeneous distribution. (ii) The bromine atoms originating from the Cu(II)Br catalyst occur in the polymerization solution. These bromine atoms diffuse less readily in the nanopores than the bromine atoms (in the initiator) introduced via the vapor. (iii) Since the quantum dots that are introduced as nanoparticles in suspension are much larger than the initiator and the catalysts molecules, it is a reasonable inference that the QDs diffuse even less readily than the vapor and the polymerization solution, thereby leading to a greater inhomogeneity. Moreover, additional inhomogeneity arises during the rinsing of excess quantum dots after infiltration. On the basis of the details explained above, we expect that the maximum and the minimum amplitudes of the lead signal vary more than for bromine.

Let us evaluate our results in light of the three main requirements that we set out with. First, we aimed for (a) nanometer resolution (b) at great depths inside 3D nanostructures. (a) At present, the resolution is determined by the size of the X-ray focus (23 nm by 37 nm, see Methods) and the step size of the spatial scan (50 nm), where the latter was in turn set by the total measurement duration. It would be advantageous to further improve the resolution as it would allow to resolve the brush thickness. A straightforward way to improve the resolution such that it is limited only by the focus size, is to increase the incident photon flux and improve the detector technology. This is exactly what is being done in the ongoing refurbishments of several large synchrotrons including ESRF. The increased coherence of the new synchrotron X-ray beams will also allow for employing smaller foci and hence a further improved resolution. (b) To probe elements deep inside nanostructures, we employed high-energy X-ray photons (17 keV) to achieve a high penetration depth. Such a high photon energy also allows for samples to be integrated on thick substrates like wafers.^40^ At the ID16A instrument used in the present study, it is also feasible to excite at an even higher photon energy of 33.5 keV, but this makes little sense since a major limitation is the absorption of the emitted X-ray fluorescence that has a fixed energy. Exciting at higher photon energy will reduce the excitation efficiency and therefore also reduce the sensitivity. Our choice of 17 keV is nearly ideal to excite all elements up to Br (absorption edge at 13.5 keV) and Pb (highest L-edge at 15.9 keV). The K-edge of Pb at 88 keV is out of reach with nanofocusing; moreover, exciting this absorption edge would result in highly inefficient excitation of other, lighter, elements.

Second, we aimed for element specific detection. Indeed, we successfully detected Br, Ga, Pb, and Cr with a high signal-to-noise ratio. For Cl and Cu, the collected data had a low signal-to-noise ratio, due to a surface contamination that was hiding the weaker bulk signal. Moreover, the signal of these low-*Z* elements at relatively low photon energy is susceptible to self-absorption by the bulky sample. The outcome could potentially be improved for these low-*Z* elements by using a confocal detection scheme^41^ or wavelength dispersive X-ray spectroscopy.^42^ Nevertheless, combining these techniques with nanotomography remains a major technical challenge. It is an interesting suggestion to design the monomer for the polymer synthesis with a heavier element as a marker, such that it is more readily detected by X-ray fluorescence.

Third, we aimed for a nondestructive probing method. Our method meets this requirement as no irreversible mechanical modifications like cleaving or milling are needed. To further confirm the integrity of our samples, we verified that we could still detect near-infrared luminescence from the PbS quantum dots after the X-ray experiments.

## Conclusions

We have performed X-ray fluorescence tomography to locate the positions of quantum dots that were infiltrated deep inside a 3D nanostructure, a photonic band gap crystal, made from silicon. This demonstration leads to opportunities in a number of applications. In nanophotonics, it is known that the fundamental local density of optical states (that controls the fluorescence of embedded emitters) has interesting properties, either maxima or minima, that do not necessarily appear at interfaces between constituent material.^20^ For instance, in ref (43), it was found that the LDOS has a maximum at low-symmetry positions in the unit cell of inverse opals. Thus, to optimally control an emitter, one has to position the emitter at these positions. Assuming that the technology to position the emitter exists (see, e.g., ref (26)), one must then probe the resulting positions to verify the functionality of the device, and this is exactly the step forward offered by the present X-ray fluorescence tomography study. In other technologies, like batteries or sensors, see e.g., ref (44), it is vital to position functional nanoparticles on the interfaces between constituent materials or phases, while the functionality or its efficiency greatly decrease if the nanoparticles are displaced. So also for these applications, X-ray fluorescence tomography can verify the intended positions to a high (nanometer) precision, and thereby greatly contribute to improved batteries and sensors.

## Methods/Experimental

### 3D Si Photonic Crystals

Our 3D photonic band gap crystals were fabricated by a CMOS-compatible fabrication process that is described in detail in refs (38), (39), and (45). As substrates, we employ Si beams (cross sections 0.5 × 0.5 mm^2^) that are chemically etched to obtain perpendicular crystal surfaces. A thin 50 nm Cr layer serves as a hard etch mask that is deposited on two adjacent surfaces of a beam. In the Cr layer, we define an etch mask in a single step on *both* faces of the beam using focused-ion beam writing with Ga ions.^39^ We perform deep reactive ion etching using the Bosch process (a time-multiplexed alternating process that alternates etching and lamination steps) to etch two perpendicular arrays of deep nanopores.^38^

In the etching step, pores are etched with SF_6_, and in the lamination step the pore walls are protected with a CF polymer layer. The radius of the nanopores etched in the silicon was designed to be *r* = 160 nm, and the lattice parameters are *a* = 680 nm and *c* = 481 nm.

Figure 1 shows a SEM image of a successfully etched photonic crystal. The overlap region of the pores entering into the *XY* surface (top) and into the *XZ* surface (bottom) mutually cross inside the beam and form the 3D cubic inverse woodpile structure that has a broad photonic band gap.^24,46^

The size and shape of the resulting 3D cubic inverse woodpile structure depends on the etch depths of both sets of pores. The deeper each set of pores is etched into the silicon, the larger the volume of the inverse woodpile structure. For this specific sample studied here, the resulting 3D volume was smaller than aimed for. The reason is that the second etching step resulted in shorter pores than the first one etched due to high amounts of fluorocarbons on the surface for the second set of pores. Therefore, the pores in the *X* direction do not cross all pores etched in the *Z* direction (see Figure 1a); therefore, at larger *X* positions there is a 2D crystal of *Z* directed pores adjacent to the 3D crystal structure near the edge of the wafer.

### Cleaning

The Cr hard mask was removed in ceric ammonium nitrate-based etchant for 60 s. The beam was intensively rinsed for 1 h with deionized (DI) water and dried in nitrogen stream.

RCA-2 cleaning (RCA-2 was developed in 1965 by Werner Kern while working for the Radio Corporation of America (RCA)) was used to remove metal residues from the samples.^47^ A glass beaker was placed on a hot plate. A total of 1500 mL of DI water was added, and while magnetically stirring, 300 mL of hydrochloric acid was slowly added. The solution was heated to 70 °C; 300 mL of hydrogen peroxide was slowly added under stirring. Once the solution reached 70 °C, the samples were added and were kept in the RCA-2 solution for 15 min. The samples were repeatedly rinsed in a water bath until a resistivity >10 MΩcm was reached, indicative of an acid-free environment.

Organic traces were removed by placing a sample in a bath of HNO_3_ (99%, room temperature) for 10 min. The sample was transferred to a second bath under the same conditions and cleaned for another 10 min and rinsed with DI water and dried under a nitrogen flow. The dried sample was transferred into a closed beaker of boiling HNO_3_ (69*%* in water, 95 °C) and cleaned for 10 min. The sample was rinsed with DI water and dried under nitrogen. Samples were placed in a clean beaker that was closed and kept on a hot plate to prevent water from entering the pores before the furnace cleaning. The samples were thermally treated in a furnace at 800 °C under 100% O_2_ for 90 min to oxidize and remove the remaining fluorocarbons (from the etching step) from the pore surface inside the silicon.

### Polymer Brush Infiltration

The polymer brush infiltration is described in two parts: first, the attachment of the initiator to the silicon beam; second, the SI-ATRP of the poly(glycidyl methacrylate) brushes.

### Chemical Vapor Deposition of the Initiator

The ATRP initiator was synthesized following Ramakrishnan et al.^48^ A piranha solution was prepared with the ratio of three parts H_2_SO_4_ to one part H_2_O_2_ (volume ratio, v/v %). The silicon beam was placed into the sample holder. The sample holder was lowered gently into the piranha solution, and the sample was cleaned for 30 min. The silicon beam was rinsed 10 times with water and ethanol. A desiccator was rinsed by vacuum pumping for 5 to 10 min. The initiator was taken from a vial using an argon purged syringe. A few droplets of initiator (30 μL) were placed inside a small plastic Petri dish that was placed in the middle of the desiccator. The Si beam was placed close to the Petri dish such that the vapor diffuses into the nanopores of the photonic crystal. The desiccator was vacuum pumped for 15 min, and the chemical vapor deposition (CVD) process proceeded for 16 h. Next, the beam was placed into a flask with 100 mL of toluene in an ultrasonication bath for 20 s to remove excess initiator. Each substrate was rinsed 10 times with ethanol and water before the substrates were dried under a nitrogen stream. The silicon beam was stored in a nitrogen box.

### Chemical Synthesis of PGMA Polymer Brushes

Glycidyl methacrylate (GMA, 10 mL, 75 mmol) monomer and 2,2′-bipyridine (BiPy, 282 mg, 1.81 mmol) were added to a mixture of water (2 mL) and methanol (8 mL).^49^ The solution was purged with argon for 30 min under continuous stirring. CuCl (72.8 mg, 0.74 mmol) and CuBr_2_ (7.8 mg, 0.035 mmol) were added to another flask and flushed with argon for 15 min. The monomer solution was transferred by a previously flushed syringe to the catalyst flask. The resulting polymerization solution was stirred for an additional 30 min under argon. The silicon beam was taken out of the nitrogen box shortly before the reaction and was placed into a reaction flask. The reaction flask was attached to a vacuum pump. Afterward the monomer solution was transferred to the reaction flask containing the silicon beam. The polymerization was allowed to proceed for 60 min. The silicon beam was cleaned extensively with acetone and water-free dimethyl sulfoxide to remove nonreacted monomer, metal complex, and remaining catalyst residues. The silicon beam was dried in a nitrogen stream to obtain the sample with the thin PGMA brush film.

### Quantum Dot Nanoparticles

We study PbS nanoparticle quantum dots, whose emission wavelengths are compatible with the Si photonic crystals; that is, the emission wavelength is longer than the Si band gap absorption edge (1100 nm). The nanoparticles were obtained in aqueous suspension from Suzhou Xingshuo Nanotech Co., Ltd. (MesoLight). From the peak of the emission spectrum measured with the dots in the original suspension at 9560 ± 60 cm^–1^, we estimated the average quantum dot diameter to be *D* = 3.47 ± 0.09 nm, using the calibration of Moreels et al.^50^ From the average diameter, we estimate that there are *N*_QD_ = 402 ± 31 lead atoms per quantum dot. The inorganic lead sulfide core of the quantum dots is covered by a poly(ethylene glycol)-amine ligand that is used to couple to a PGMA polymer layer on the silicon photonic crystal.

**Figure 6:**
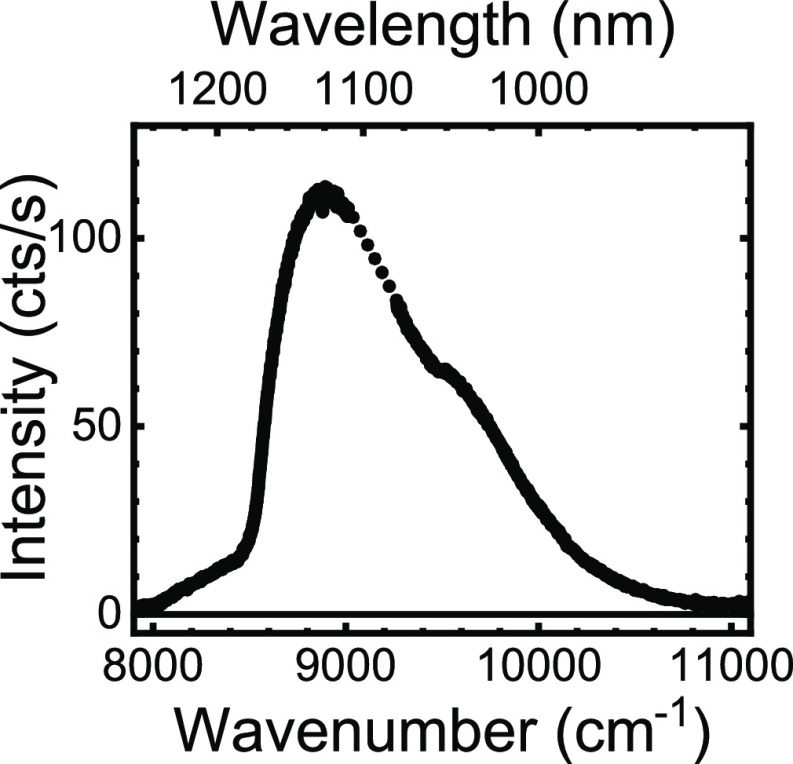
Optical emission spectrum of the PbS quantum dots on a flat Si substrate next to the photonic crystals, taken after the synchrotron experiments (solid curve). Between 9013 and 9263 cm^–1^, the detector pixels are dead, therefore we linearly interpolate (dotted).

Figure 6 shows the optical emission spectrum of the quantum dots taken after the synchrotron experiments with the setup described in ref (3). The spectrum has a peak near 9000 cm^–1^ (or λ = 1120 nm). Since the spectrum is collected from dots on the flat Si substrate, the spectrum is red-shifted compared to the dots in suspension, which is reasonable due to the higher dielectric constant of the silicon compared to water. Since the quantum dots reveal substantial excitonic intensity, we conclude that the X-ray experiments have left sufficient dots intact for further nanophotonic experimental studies.

### Synchrotron X-ray Fluorescence (SXRF) Tomography

**Figure 7:**
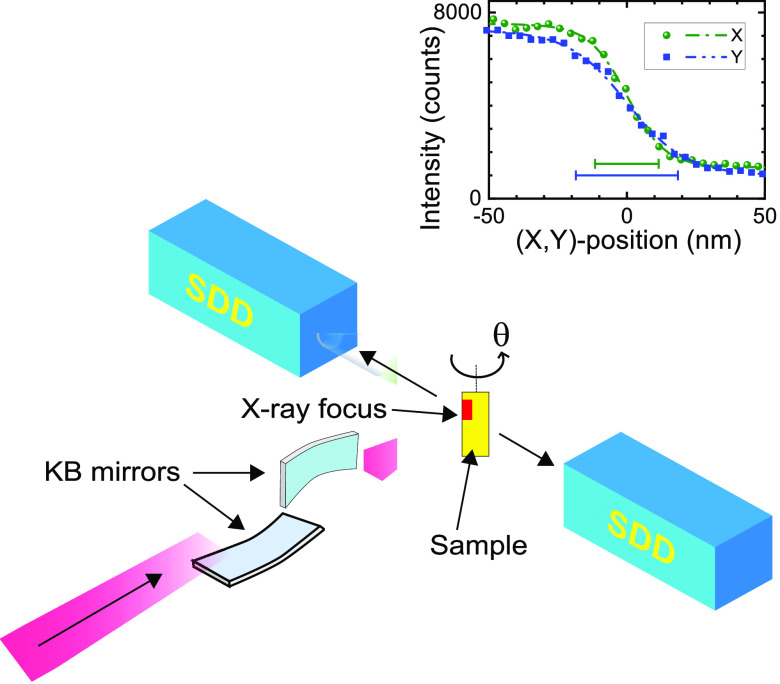
Schematic of the X-ray fluorescence setup. The X-ray beam arrives from bottom left and is focused by Kirkpatrick–Baez (KB) optics on the sample. The fluorescent light is detected by two silicon drift detectors (SDD) placed at ±90° with respect to the incident beam. The sample is rotated angle θ about the vertical axis. The inset presents the knife edge measurements of the excitation beam (intensity versus *X* or *Y* position) for a calibration sample. The resulting full widths at half-maximum (fwhm) are Δ*X* = 23 nm and Δ*Y* = 37 nm.

Figure 7 shows the setup to perform X-ray fluorescence nanotomography at ESRF beamline ID16A.^51,52^ X-ray fluorescence is excited by the incident beam (fwhm widths Δ*X* = 23 nm and Δ*Y* = 37 nm) with a photon energy set to 17 keV with a 1% relative bandwidth. The X-ray beam is focused by multilayer coated Kirkpatrick–Baez (KB) optics onto the sample. At every sample orientation θ, the exciting X-ray focus is continuously scanned through the crystal in the horizontal direction and stepwise in the vertical direction to image the whole crystal. This corresponds to maps with 240 × 240 pixels (50 nm size), corresponding to a total area of 12 × 12 μm^2^. At each sample position (*x*_*i*_,*y*_*j*_) the X-ray fluorescence photons are collected by two six-element silicon drift diode detectors with an energy resolution of 130 eV at 5 keV photon energy that are placed at ±90° with respect to the incident beam; their readings are combined into an energy-resolved spectrum *I*(*E*,θ,*x*_*i*_,*y*_*j*_).

The chemical species that can be detected depend on the constituent elements and their fluorescence spectra. Whether such an element can be detected depends on whether its transition fits in the available spectral range, as shown in Figure 2. The available range in turn depends on factors like the excitation energy (here 17 keV) and a possibly present substrate or matrix (here the silicon backbone of the photonic band gap crystal) that emits intense peaks (here at 1.74 keV).

We note that there are fundamental limitations to the detection based on the involved chemical species and on their combination. On one hand, heavy elements are more readily detected since the photon energy of their fluorescence is sufficiently high to be detected, see Figure 2. In our case, Pb peaks are strong and clearly detected at 10.5 and 12.5 keV, whereas the Cl peak (2.6 keV) at a low photon energy is swamped by S and Pb transitions. On the other hand, if combinations of elements yield overlapping fluorescence peaks, this also affects their detection, e.g., in our case the S K_α_ peak (near 2.3 keV) cannot be properly modeled since it overlaps with the Pb M_α_ transition. Each detection pixel has an area of 50 × 50 nm^2^. The integration time at each sample position was set to 50 ms. The flux of 7.5 × 10^9^ photons/s is kept far below the maximum flux to prevent detector saturation. Hence, collecting one 240 × 240 pixel fluorescence image took about 1 h, and collecting a full tomographic scan over 17 angles took about 20 h, excluding the time for alignment. In view of the current measurement time and data analysis, the SXRF method is not yet suited to iteratively optimize the infiltration.

The sensitivity of the method is estimated to be 0.4 ppm for the relative density of Pb atoms (of the quantum dots) versus Si atoms (of the photonic crystal backbone.) We obtain this sensitivity by first calculating the total number of lead atoms for the entire crystal volume from our data (integrating Figure 5e). Second, from that value we estimate the density of lead atoms per cubic micron. Third, we compare that value with our estimation of the silicon atoms per micron. Hence we detect 24 000 Pb atoms compared to 50 × 10^9^ Si atoms = 24 000/50 × 10^9^ = 0.4 ppm.

### Volume Reconstruction

**Figure 8:**
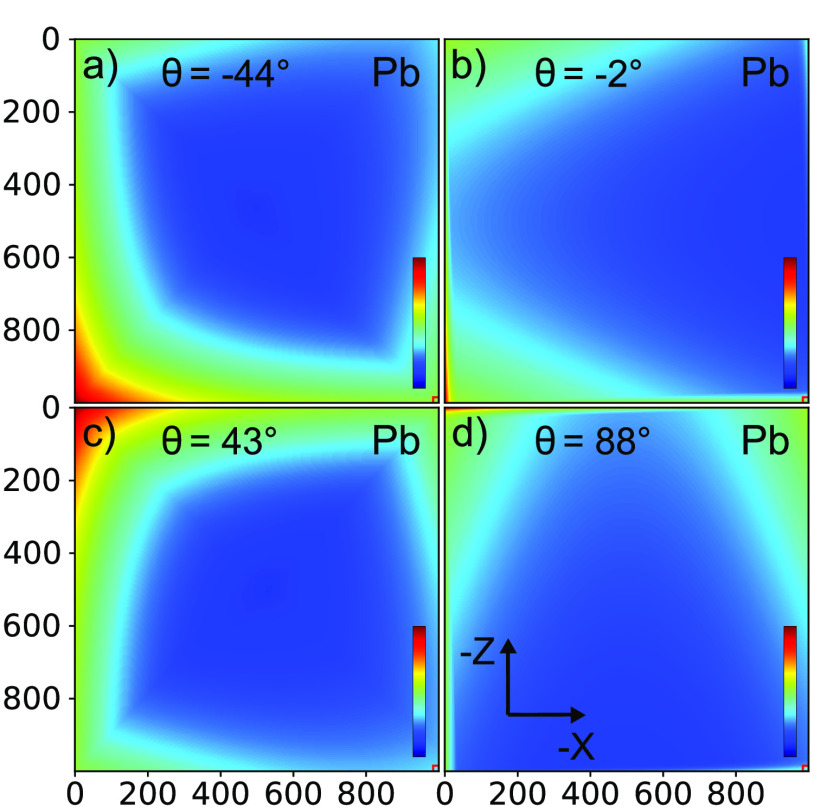
Attenuation maps of lead fluorescence as a function of atomic position inside the Si beam. In this representation, the Si-beam orientation is fixed with the photonic crystal (small square) at bottom right, whereas the incident X-ray beam orientation is varied: (a) θ = −47° beam incident from bottom left, (b) θ = −2° beam from left, (c) θ = 43° beam from top left, (d) θ = 88° beam from top. All color bars run from 0 to 1.

In the volume reconstruction, the mass density distribution is reconstructed from the angular projections through tomographic reconstruction. In this case, the radon transform is severely undersampled and iterative, algebraic reconstruction methods have to be used. The volume reconstruction consists essentially of three steps: (A) a drift correction on the projections, (B) the tomographic reconstruction, (C) corrections for self-absorption.

(A) *Drift correction*: Due to the very long measurement times, lateral drifts occur that have to be corrected. The alignment was carried out with ESRF in-house software using the GNU Octave (http://www.octave.org) programming environment and the TomoJ plugin of the public domain image analysis program ImageJ (http://rsbweb.nih.gov/ij). The vertical and the horizontal drifts were separately corrected using the preservation of the vertical mass profile under rotation^53^ and the landmarks refinement of the TomoJ plugin, respectively. Gallium, the element that exactly defines the position of the pores, was used as the reference element for the drift correction. During the writing of the etch mask by focused-ion beam (FIB), the chromium hard mask is bombarded with gallium ions. That means that gallium is present on the surface at the locations where the pores are written. We used four landmarks on the elbow-shaped marker as suitable reference points. The data were corrected in such a way that the location of a specific landmark describes a sinusoidal trajectory for the different rotation angles. The drifts, as determined for gallium, were then compensated for on all elements using bicubic interpolation.

(B) *Volume reconstruction*: Since the number of successfully collected projections was only 17, a regularized iterative tomographic reconstruction method was chosen. We used the Total Variation Minimization (TVM) method with a positivity constraint as implemented in the PyHST software developed at ESRF.^54^ The parameters for the regularization and the number of iterations were adjusted visually for the best outcome of the reconstruction. The final conditions of the reconstruction were as follows: 300 iterations of the Chambolle–Pock optimization algorithm without preconditioning and with positivity constraint and a TV regularization parameter of 10^–5^ and 10^–3^ respectively for Ga and for Br and Pb. The sample substrate induces the tomography to be local in nature. To compensate for this, the projections were padded horizontally by extension with the last value, and the support of the volume reconstruction was taken to be a cylinder with 240 pixels height and 300 pixels diameter, i.e., slightly wider than the original projections.

(C) *Self-absorption*: To estimate the effect of the self-absorption, we used the package *PyCorrectedEmissionCT*.^55^ Attenuations were included of both the incident 17 keV X-ray beam and of the emitted X-ray fluorescence along its path to the detectors. We modeled the sample using a Si beam with a 500 × 500 μm^2^ cross-section and in one corner a 8 × 8 μm^2^ region with half the density of silicon representing the photonic crystal. For each element under study, a weighted average energy of the X-ray fluorescence was considered, and the corresponding attenuation coefficient was calculated using the Xraylib library.^56^ Next, attenuation maps per element were calculated for all angles of the tomography scan. As an illustration, Figure 8 shows the result for the Pb fluorescence at four angles, which show that the angle-dependent attenuation is fairly constant. Therefore, and to keep the tomographic inversion tractable, we chose to compensate for the self-absorption by scaling-up all tomography by the inverse of the mean attenuation, averaged spatially over the photonic crystal and averaged over all tomography angles. This means attenuation amounts to 0.168, 0.404, 0.415, 0.445, and 0.439 respectively for Cl, Cu, Ga, Br, and Pb.

A preprint of this study is available on the ChemRxiv preprint server.^57^

## Supporting Information Available

(S1) Scheme of the chemical synthesis; (S2) *XZ* cross sections of Ga versus *Y*; (S3) *XZ* cross sections of Br versus *Y*; (S4) *XZ* cross sections of Pb versus *Y*; (S5) projections of Ga versus θ; (S6) projections of Br versus θ; (S7) attenuation for Ga for four angles; (S8) attenuation for Br for four angles; (S9) attenuation for Cu for four angles; (S10) attenuation for Cl for four angles; (S11) 3D renderings of Ga, Br, and Pb in the 3D nanopore structure (PDF)
Movie S1: color animation of all cross sections for gallium (AVI)
Movie S2: color animation of all cross sections for bromide (AVI)
Movie S3: color animation for all cross sections for lead (AVI)

## Supplementary Material

nn1c06915_si_001.pdf

nn1c06915_si_002.avi

nn1c06915_si_003.avi

nn1c06915_si_004.avi
