# Supplementary material for: Targeted Positioning of Quantum Dots Inside 3D Silicon Photonic Crystals Revealed by Synchrotron X-ray Fluorescence Tomography
Source: ACS Nano. 2022 Feb 21;16(3):3674–83. doi: 10.1021/acsnano.1c06915 (PMC8945387; doi:10.1021/acsnano.1c06915)

Supporting Information: Targeted positioning of quantum dots inside 3D silicon photonic crystals revealed by synchrotron X-ray fluorescence tomography

Andreas S. Schulz,[†,‡,¶] Cornelis A. M. Harteveld,[†] G. Julius Vancso,[¶] Jurriaan Huskens,[‡] Peter Cloetens,[*,§] and Willem L. Vos[*,†]

†*Complex Photonic Systems (COPS), MESA+ Institute for Nanotechnology, University of Twente, P.O. Box 217, 7500 AE Enschede, The Netherlands*

‡*Molecular Nanofabrication (MNF), MESA+ Institute for Nanotechnology, University of Twente, P.O. Box 217, 7500 AE Enschede, The Netherlands*

¶*Materials Science and Technology of Polymers (MTP), MESA+ Institute for Nanotechnology, University of Twente, P.O. Box 217, 7500 AE Enschede, The Netherlands*

§*ESRF-The European Synchrotron, CS40220, 38043 Grenoble, France*

E-mail: cloetens@esrf.eu; w.l.vos@utwente.nl

Supporting Information Available

This supplementary features 11, details of the chemical synthesis, 2) video animations of all slices of the three-dimensional (3D) data sets of various elements, 3) projection maps of several elements, 4) attenuation maps as an important correction step, and 5) 3D renderings of several elements.

1 Scheme of the chemical synthesis

The chemical synthesis is described in three parts: firstly, the attachment of the initiator to the silicon beam, secondly, the SI-ATRP of the poly(glycidyl methacrylate) brushes, and thirdly the coupling of the quantum dots, see scheme S1 below.

S1 Chemical synthesis 'S1_scheme_chemistry.pdf':

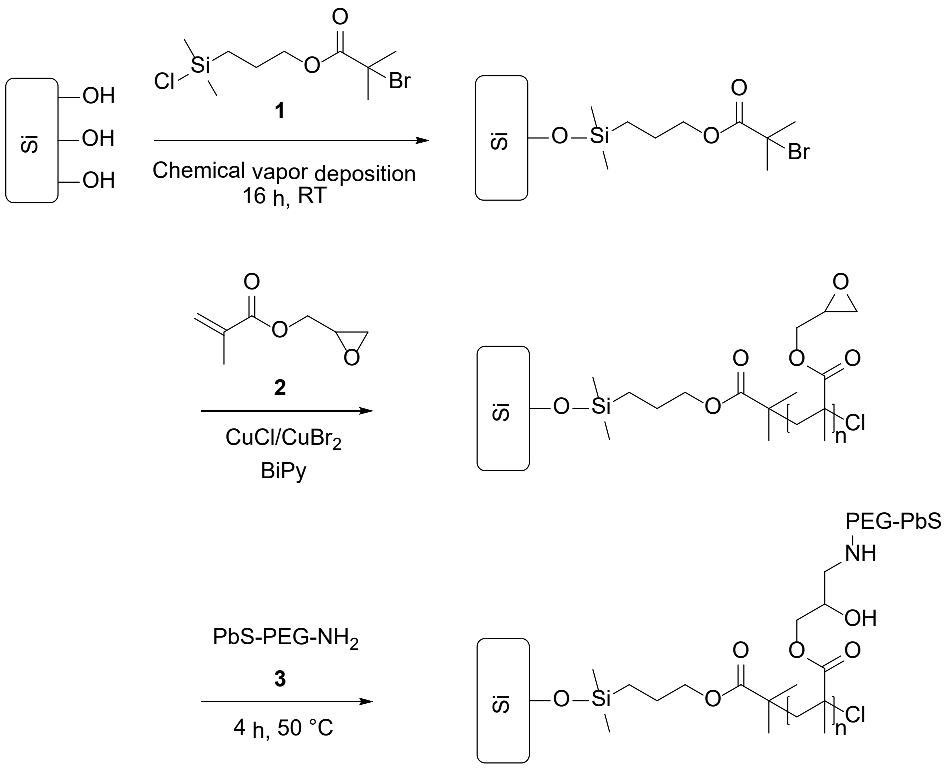

2 Multimedia Files

In view of the richness of the 3D reconstructions, we provide here movie animations of cross-sections through the 3D data volumes.

S2 Animation 'S2_Ga-K_xz.avi': Colour animation of all cross-sections for gallium, of which 2 slices are shown in Fig. 4A-B of the main manuscript. The animation shows

the 3D reconstructed sample volume for all cross-section for the XZ-direction. Below is a still at 0:03, where the label top left is the slice number (here 101) and the label top right shows the highest Ga density found in the entire slice.

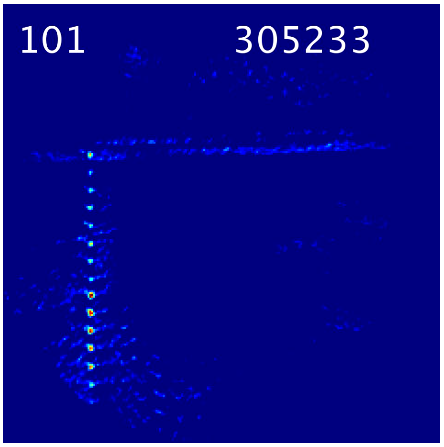

S3 Animation 'S3_Br-K_xz.avi': Colour animation of all cross-sections for bromide, of which 2 slices are shown in Fig. 4C-D of the main manuscript. The animation shows the 3D reconstructed sample volume for all cross-section for the XZ-direction. Below is a still at 0:03, where the label top left is the slice number (here 131) and the label top right shows the highest Br density in the entire slice.

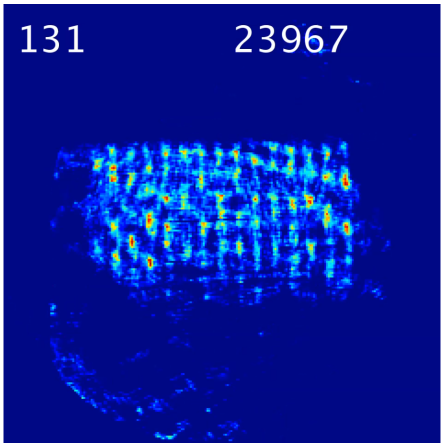

S4 Animation 'S4_Pb-L_xz.avi': Colour animation of all cross-sections for lead, of which 2 slices are shown in Fig. 4C-D of the main manuscript. The animation shows the 3D

reconstructed sample volume for all cross-section for the XZ-direction. Below is a still at 0:02, where the label top left is the slice number(here 70) and the label top right shows the highest Pb density found in the entire slice.

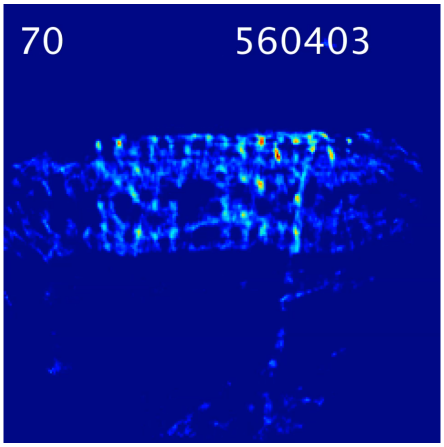

3 Projection maps

We present here projection maps for the elements gallium and bromine.

S5 Animation 'S5_att_cor_Ga_projections_4in1.pdf':

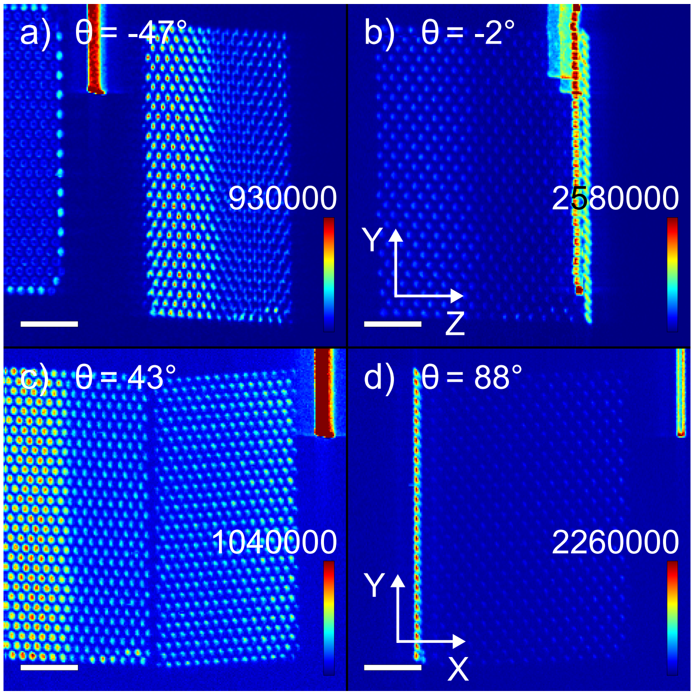

Two of these Ga projections are shown in the main manuscript as Fig. 3 (a,b).

S6 Animation 'S6_att_cor_Br_projections_4in1.pdf':

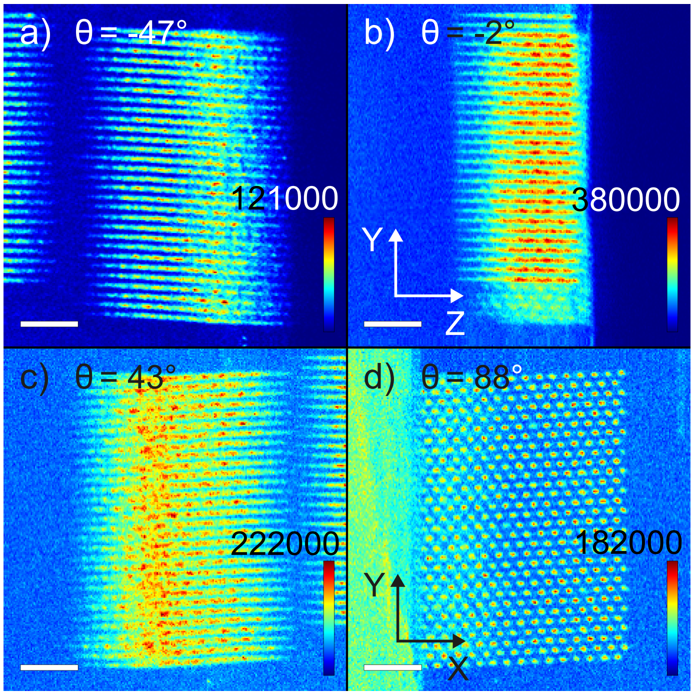

Two of these Br projections are shown in the main manuscript as Fig. 3 (c,d).

4 Attenuation maps

Here we present the attenuation maps for the elements gallium, bromine, copper, and chlorine.

S7 Animation 'S7_att_Ga_4angles.pdf':

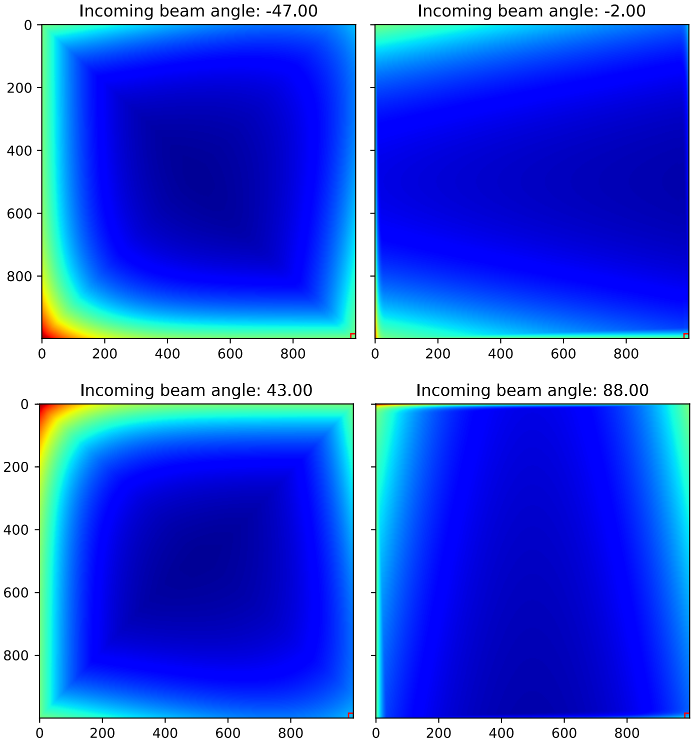

S8 Animation 'S8_att_Br_4angles.pdf':

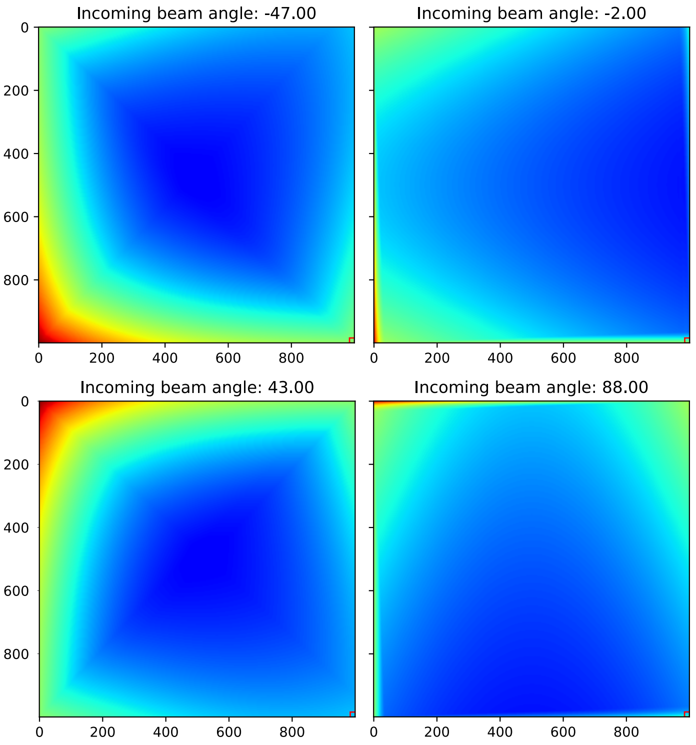

S9 Animation 'S9_att_Cu_4angles.pdf':

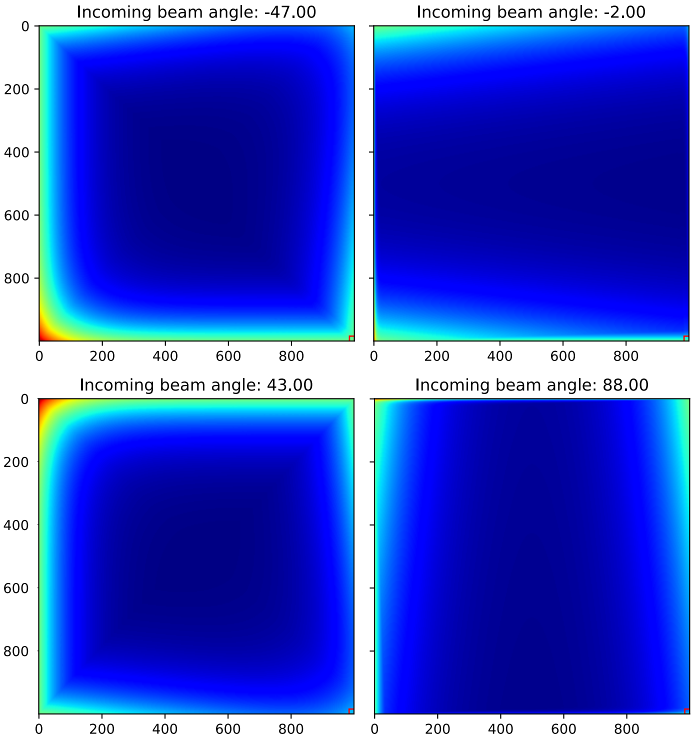

S10 Animation 'S10_att_Cl_4angles.pdf':

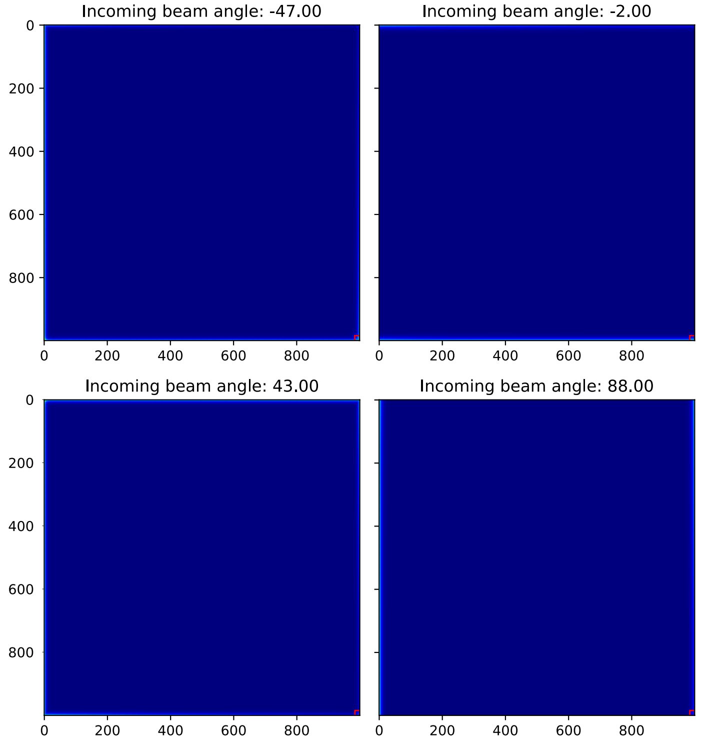

5 3D rendering

Here we present the 3D renderings for the elements gallium (light blue), bromine (yellow), and lead (red).

S11 Animation 'S11_Ga_cyan_Br_orange_Pb_red_pores_filled_with_sharp_lines_Pb.pdf':

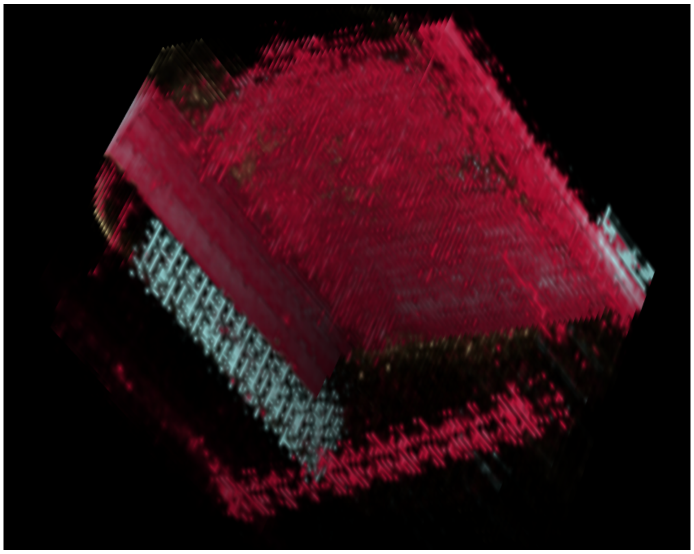

Supplement: Supplementary file 1 — nn1c06915_si_001.pdf [file nn1c06915_si_001.pdf]
